# Hollow CuS nanocubes enhance serum metabolic profiles for rapid diagnosis and severity grading of traumatic brain injury

**DOI:** 10.1016/j.mtbio.2025.102586

**Published:** 2025-11-21

**Authors:** Lei Shi, Ping Yuan, Jiaxin Hou, Junxi Pan, Kejia Cao, Yanhui Wang, Si Cheng, Xuting Shen, Yongli Yang, Nengrui Guo, Yizhen Pan, Rongxin Li, Weian Yuan, Lijun Bai

**Affiliations:** a The Key Laboratory of Biomedical Information Engineering, Ministry of Education, Department of Biomedical Engineering, School of Life Science and Technology, Xi'an Jiaotong University, Xi'an, Shaanxi, 710049, China; b Department of Clinical Laboratory, Shuguang Hospital Affiliated to Shanghai University of Chinese Traditional Medicine, Shanghai, 201203, China; c Department of Cardio-Pulmonary Circulation, Innovation and Incubation Center (IIC), Shanghai Pulmonary Hospital, School of Medicine, Tongji University, Shanghai, China; d School of Chemistry and Molecular Engineering, East China Normal University, Shanghai, 200241, China; e Department of Clinical Laboratory, The First Affiliated Hospital of Kunming Medical University & Yunnan Province Clinical Research Center for Laboratory Medicine, Kunming, 650032, China; f Institute of Translational Medicine, Shanghai Jiao Tong University, Shanghai, 200241, China; g Clinical Research Center, Shuguang Hospital Affiliated to Shanghai University of Traditional Chinese Medicine, Shanghai, 201203, China

## Abstract

Traumatic brain injury (TBI) remains a significant global health burden and demands rapid, objective diagnostics that work outside imaging suites and intensive lab workflows. Here, we developed a copper sulfide (CuS) nanocube–assisted laser desorption/ionization mass spectrometry (LDI-MS) platform to acquire serum metabolic profiles (SMPs) with high sensitivity, low background in the low-m/z region, and excellent reproducibility for TBI diagnosis and severity grading. Using this platform, we profiled serum from individuals with TBI and healthy controls and achieved high-accuracy discrimination of TBI from Healthy Controls (HCs) (AUC = 0.999 in both training and independent test sets). The model further enabled reliable grading between mild and severe TBI. Feature selection yielded a panel of discriminative *m*/*z* signals that reflect systemic metabolic reprogramming after brain injury. Pathway analysis indicated perturbations in central carbon and amino-acid metabolism, consistent with altered energy production and nitrogen handling in TBI. In conclusion, CuS-assisted LDI-MS and SMP-based modeling provide a fast, reproducible, and mechanistically grounded route toward deployable serum tests for TBI diagnosis and staging.

## Graphical abstract

We develop a hollow CuS nanotubes-assisited MALDI MS platform for serum metabolite profiling to diagnosis and severity grading of traumatic brain injury. The platform enable rapid extraction of 320 serum metabolic profiles and achieved superior diagnostic and grading performance. This work provides a practical approach to rapid serum metabolomics, which can inform triage and severity staging in time-critical clinical contexts.

**Figure:**
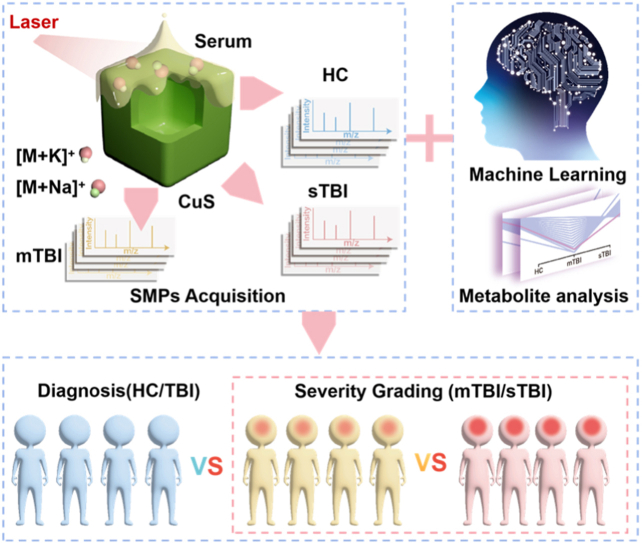

## 1 Introduction

Traumatic brain injury (TBI) remains a global public health crisis from sports and traffic incidents to falls and combat, with an estimated 50–60 million new cases annually and a worldwide economic burden of approximately $400 billion [1]. Despite its prevalence and significance for public health systems, TBI remains challenging to diagnose and stage rapidly, objectively, and at scale. Neuroimaging modalities such as computed tomography (CT) and magnetic resonance imaging (MRI) are indispensable for detecting focal lesions [2], hemorrhage, and structural abnormalities; yet, they require specialized infrastructure and trained personnel [3]. Moreover, severity grading with clinical scales—most notably the Glasgow Coma Scale (GCS)—provides valuable bedside grading but only partially reflects the underlying biology; two patients with identical GCS scores can diverge markedly in trajectory and outcome [4]. Consequently, the clinical community continues to seek molecular tools that can complement imaging and clinical assessment, enabling earlier triage, better risk grading, and more informed resource allocation [5].

Blood-based measurements are attractive in this context because TBI perturbs brain energy metabolism, redox balance, lipid remodeling, and neuroinflammatory signaling. These processes generate small molecules that can traverse a compromised blood–brain barrier (BBB) or otherwise appear in the circulation [6]. Serum and plasma offer practical advantages because they are accessible in virtually any clinical environment, compatible with repeated sampling, and amenable to workflows that fit urgent decision timelines [7]. Metabolomics—the comprehensive measurement of small-molecular-weight biochemicals—provides a particularly sensitive lens on bioenergetics, membrane dynamics, and neurotransmission-linked pathways [8]. In principle, a well-designed metabolomic readout can convert complex, spatially heterogeneous brain pathology into a robust, systemic fingerprint.

Mass spectrometry (MS) is the workhorse for metabolomics because it combines sensitivity with chemical breadth [9]. However, conventional liquid chromatography–MS (LC–MS) workflows require extraction, cleanup, and time-consuming separations, and thus they limit throughput and delay results, especially in emergency or resource-limited settings [10]. In contrast, laser desorption/ionization MS (LDI–MS) bypasses chromatographic separation and can analyze microliter-scale biofluids within minutes [11]. Nonetheless, its analytical performance depends critically on the solid matrix, which must harvest light, convert it to heat and charge, and mediate desorption/ionization. In most implementations, organic matrices such as α-cyano-4-hydroxycinnamic acid (CHCA) or 2,5-dihydroxybenzoic acid (DHB) are employed [12]. However, these matrices contribute intense chemical backgrounds and abundant matrix–cluster ions below *m*/*z* ≈ 500. Moreover, shot-to-shot variability in ion formation can inflate coefficients of variation (CVs), thereby eroding reproducibility—an unacceptable weakness for clinical translation [13].

Rationally engineered inorganic nanomaterials can substantially improve LDI performance for metabolite profiling [14]. By replacing organic matrices with optically and electronically favorable surfaces, inorganic nanomaterials can minimize low-mass chemical noise while enhancing energy harvesting and charge transfer at the analyte interface [15]. Ideal materials absorb strongly at the laser wavelength, concentrate electromagnetic fields at nanoscale features, confine thermal energy at the surface for efficient desorption, and facilitate ion formation without fragmenting themselves into the low-m/z window [16]. Metals, oxides, carbons, and chalcogenides have all been explored, yet identifying a composition–morphology combination that delivers strong signals, high reproducibility, and easy manufacturing remains an active engineering challenge [17]. Copper sulfide (CuS) is a desirable candidate for LDI. Hexagonal covellite-phase CuS is intrinsically p-type, with copper vacancies that generate high hole concentrations. This defect-rich electronic structure supports efficient photothermal conversion and plasmon-like absorption extending into the visible/near-IR, while retaining substantial absorption at 355 nm (a standard Nd: YAG MALDI wavelength) [18].

**Fig. 1:**
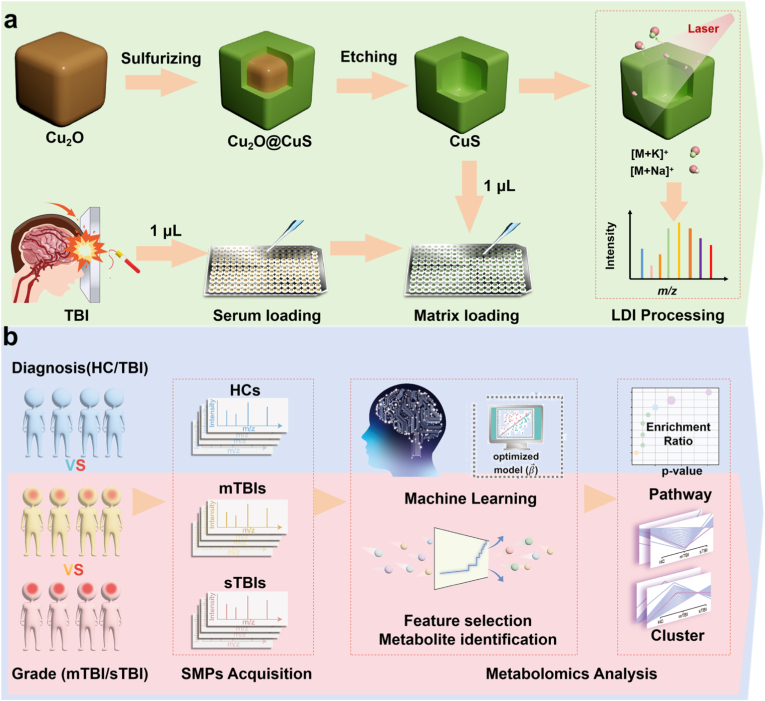
Schematic workflow for hollow CuS nanocubes that enhance serum metabolic profiles for the rapid diagnosis and grading of traumatic brain injury (TBI). a) Schematic workflow for the extraction of metabolic profiles in serum by CuS-assisted laser desorption/ionization mass spectrometry (LDI-MS). CuS exhibits good conductivity for enhanced ionization and microscale surface roughness, providing stability for the effective LDI of metabolites. Only Na^+^- and K^+^- adducted metabolites can be selectively detected with the coexistence of high concentration of peptides and proteins, consuming ∼1 μL of serum with Nd: YAG laser (355 nm); **b**) Diagnostic workflow for TBI patients (TBIs) and Healthy Controls (HCs), including data processing, machine learning of serum metabolic profiles (SMPs), and the selection of potential biomarkers for TBI diagnosis.

Here, we introduce a CuS-assisted LDI-MS platform for the rapid, high-throughput acquisition of serum metabolic profiles (SMPs) (Fig. 1a). We synthesize faceted, hollow CuS nanoparticles by sulfurizing Cu_2_O cubes followed by core etching, yielding phase-pure hexagonal CuS with strong 355-nm absorbance. Compared with CHCA/DHB and CuO controls, CuS boosts signal intensities of representative metabolites while maintaining a clean low-m/z window. The workflow requires ∼100 nL of serum co-spotted with ∼1 μg CuS, achieves <30 s acquisition per sample, and scales to 384-spot chips, reducing batch effects. To test the clinical utility, we profiled serum samples from 177 TBI patients and 143 healthy controls, applied standard preprocessing (baseline correction and normalization), and evaluated multiple machine-learning models under repeated cross-validation (Fig. 1b). Logistic regression (LR) emerged as the most parsimonious and best-performing classifier. We then derived compact diagnostic and severity-grading panels, annotated key features and interrogated pathway-level changes using KEGG enrichment. In summary, this work combines rational materials design and ultrafast analysis to provide an objective and scalable workflow for TBI assessment. We provide both a mechanistic foundation and a practical approach to rapid serum metabolomics, which can inform triage and severity staging in time-critical clinical contexts.

## 2 Experimental section

### 2.1 Chemicals and reagents

The chemicals and reagents in this work included those used in the two respects: (1) the hollow CuS nanocubes synthesis; (2) the performance evaluation for the LDI MS. In the CuS synthesis, Copper sulfate pentahydrate (CuSO_4_·5H_2_O, AR 99.9 %), Sodium citrate dihydrate (C_6_H_5_Na_3_O_7_·2H_2_O, AR 99.0 %), Sodium hydroxide (NaOH, AR 97.0 %), Ascorbic acid (C_6_H_8_O_6_, AR 99.0 %), Sodium Sulfide (Na_2_S, AR 99.9 %), Sodium Thiosulfate (NaS_2_O_3_, PT 99.9 %) were ordered from Sinopharm Chemical Reagent Beijing Co. Ltd. (Beijing, China). In the performance evaluation for the LDI-MS, 2,5-dihydroxybenzoic acid (DHB, 99 %), -cyano-4-hydroxy-cinnamic acid (CHCA, 99 %), and bovine serum albumin (98 %) were ordered from Sigma-Aldrich (St. Louis, MO, USA). Sucrose (Suc, 99.5 %), glutamic acid (Glu, 99 %), proline (Pro, 99 %), and mannitol (Man, 97 %) were purchased from Adamas (Shanghai, China). Trifluoroacetic acid (TFA, 99.5 %) and acetonitrile (ACN, 99.9 %) were obtained from Adamas (Shanghai, China). Besides, the experiment water used in this work was prepared by the ultrapure water system (18.2 MΩ cm, Milli-Q, Millipore, GmbH).

### 2.2 Synthesis of hollow CuS nanocubes

#### 2.2.1 Preparation of Cu_2_O nanocubes

Employing the same procedure as before, 0.375 g CuSO_4_·5H_2_O and 0.147 g of Sodium citrate dihydrate were dissolved in 80 mL of deionized water (DI) under stirring at room temperature. Subsequently, 20 mL of NaOH solution (1 g in 20 mL DI) and 50 mL of ascorbic acid solution (0.264 g in 50 mL DI) were added to the mixture. The resulting solution was stirred for 3 min and aged for 1 h. The Cu_2_O nancubes were collected by centrifugation, followed by washing and vacuum drying at 60 °C overnight.

#### 2.2.2 Preparation of Cu_2_O@CuS and CuS

40 mL of an aqueous sodium sulfide (Na_2_S) solution (0.06 g in 40 mL DI) was added to 60 mL of aqueous Cu_2_O solution (0.1 g in 60 mL DI). The mixture was stirred for 30 min. The Cu_2_O@CuS was then collected by centrifugation and washed.

The obtained Cu_2_O@CuS was dispersed in a 40 mL mixed solvent of ethanol/water (v/v = 1/1). Then, 8 mL of Na_2_S_2_O_3_ solution (1.264 g in 8 mL DI) was added in the above suspension, and the mixture was stirred for 30 min to allow for etching. The final product, hollow CuS, was collected by centrifugation.

### 2.3 Collection of serum samples

This research was carried out in compliance with the principles outlined in the Declaration of Helsinki and was approved by the EC of Shuguang Hospital affiliated to Shanghai University of Traditional Chinese Medicine, Shanghai, China (No. 2024-1496-079-01). Clinical data and serum samples used in this research were collected from the Shuguang Hospital. Before recruitment, all subjects in the Shuguang Hospital signed informed consent forms for the use of their clinical data and serum samples.

Serum samples were harvested according to the standard clinical protocols. Briefly, the blood samples were collected by venipuncture into lithium heparin vacutainer tubes, and centrifuged at 1800 g for 10 min. After centrifugation, aliquots of serum were stored at −80 °C until further analysis.

### 2.4 LDI MS detection and analysis

The samples were either 10-fold water dilution of serum or standard metabolites at 1 mg/mL. The matrices were 1 mg/mL of the CuS (dispersed in water), 10 mg/mL of DHB in TA30 (30 % ACN/70 % water, 0.1 % TFA), or saturation concentration of CHCA in TA30. In the conventional LDI experiment, 1 μL of the analyte or serum solution was spotted under ambient conditions onto a 384-spot polished steel target to form a thin layer. Subsequently, 1 μL of an aqueous matrix solution (1 mg/mL) was deposited onto the crystallized analyte. LDI mass spectra were acquired in positive-ion reflectron mode on an Autoflex LDI-TOF/TOF mass spectrometer (Bruker) equipped with a 355 nm Nd: YAG smartbeam laser, using delayed extraction, a 1 kHz laser repetition rate, and an acceleration voltage of 20 kV. The LDI-MS extraction delay was optimized to 150 ns? For each spectrum, 2000 laser shots were accumulated. Unless otherwise noted, five independent replicates were collected for each sample, and raw spectra were used without any smoothing. To correct the mass axis and ensure data reliability, a solution of mixed standards was tested at a regular interval. To assess tolerance to matrix interferences, a mixed solution of the standard small molecules was combined with either salt (NaCl, 0.5 M) or protein (BSA, 5 mg/mL), and subsequently analyzed. CuS nanocubes were used as the matrix at a concentration of 1.0 mg/mL.

### 2.5 Statistical analysis

The pre-processing of the raw data including peak extraction, peak alignment, normalization, and standardization was achieved by Python (version 3.8.0). The two-tailed *t*-test and one-way analysis of variance (including Tukey post-hoc tests) were used to examine the significance of differences in two-group mean comparisons and multiple-group mean comparisons, respectively (significance level was set at 0.05). The χ2 test was used to determine the significance of sex differences (sex assigned at birth) in the cohort (significance level was set at 0.05). The p-values for those were calculated by GraphPad Prism 10.0, SPSS v22.0. The power analysis, the dimensionality reduction chemometric analysis, and the unsupervised clustering for discriminating the SMPs of different grade TBI were performed by MetaboAnalyst (https://www.metaboanalyst.ca/). The machine learning of serum metabolic profiles, including model construction, feature selection and clustering analysis was conducted using logistic regression algorithm is also supported by Python (version 3.8.0). We Feature-selection workflow and panel construction were implemented through the following workflow. Firstly, Logistic regression (LR) was fitted to the training set. Features were retained if P < 0.05 (Wald test) and |fold change| > 1.5 (HC vs TBI). Retained features were ranked by the absolute standardized LR coefficient; the top 20 were kept for panel construction. Then, we enumerated all combinations of these 20 candidates with different combination, re-fitted LR for each combination, and computed AUC. The highest-AUC combination was selected as the final diagnostic panel. In case of ties, we prioritized models with better calibration and lower inter-feature correlation. The final panel was then assessed on the held-out test set. Four clusters were obtained by performing a soft clustering analysis on the 320 SMPs using a home-built R script based on the Mfuzz package in R.

### 2.6 Identification of potential biomarker

A three-step procedure was implemented to ensure accurate biomarker identification. Firstly, the differential *m*/*z* features were selected as potential biomarkers based on the contribution of each *m*/*z* feature and p-value (p < 0.05). Secondly, accurate mass measurements of these features were conducted with the FT-ICR-MS (<1 ppm). Thirdly, the biomarker compounds were identified by matching with the Human Metabolome Database (HMDB). Finally, LC-MS/MS analysis to confirm the chemical identity of these critical diagnostic features. For sample preparation, 400 μL of methanol/acetonitrile (50/50, v/v) solution was added to 100 μL of the representative serum sample (equally mixing six samples per group) and was vortexed for 1 min. Next, the mixture was placed at −20 °C for 2 h, and then centrifuged for 20 min at 13,000 rpm. The supernatant was collected, dried by centrifugation at 4 °C, and redissolved in 150 μL of methanol/water (30/70, v/v) for LC-MS/MS analysis. And a ZenoTOF 7600 (SCIEX, USA) UPLC-QTOF system was employed to carry out the LC-MS/MS analysis. The MS range was set at 50–1000 Da, and the MS/MS range was set at 50–500 Da. An IDA acquisition mode was employed with a maximum of 25 of MS/MS experiments per cycle. The MS/MS experiments were performed using CID fragmentation with a collision energy of 35 V for positive mode and −35 V for negative mode, and the energy spread was set at 15 V.

## 3 Results and discussion

### 3.1 Performance of CuS-enhanced LDI-MS for metabolite detection

**Fig. 2:**
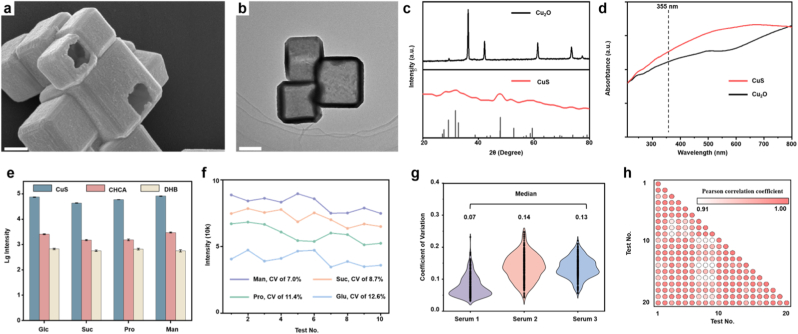
Performance of CuS-enhanced LDI-MS for metabolite detection. a) SEM image, **b**) TEM image, **c**) XRD pattern of CuS and CuO, **d**) The ultraviolet visible (UV–vis) absorption spectra of CuS and CuO, **e**) Comparison of the LDI-MS intensity of the four metabolite standards among the use of CuS, α-cyano-4-hydroxy-cinnamic acid (CHCA), and 2,5-dihydroxybenzoic acid (DHB). The standards included sucrose (Suc), glutamic acid (Glu), proline (Pro), and mannitol (Man). The data represented ten independent repeated tests and was expressed as the mean ± standard error. **(f)** CVs of the 265 *m*/*z* features detected from the representative serum sample by the MALDI-MS in the 30 independent from 3 serum repeated detections. The median CVs were 7 %, 14 %, and 13 %. **(g)** CV distribution of intensities for the apparent signals extracted through data preprocessing using three serum samples by five independent tests. **(h)** Pearson correlation coefficients among the 30 independent repeated detections for the representative serum sample.

To record the SMPs of HCs and TBIs, we constructed a high-performance LDI-MS platform based on the CuS nanoparticles for metabolic analysis. Hollow CuS nanocubes is synthesized using a multi-step liquid-phase deposition method. Briefly, CuS was prepared by first synthesizing Cu_2_O nanocubes (Figs. S1–S2) via alkaline ascorbate reduction of CuSO_4_, then sulfurizing them with Na_2_S to form Cu_2_O@CuS, and finally etching the Cu_2_O core in a 1:1H_2_O/EtOH solution with Na_2_S_2_O_3_ to yield hollow CuS. Scanning electron microscopy (SEM) image shows well-defined faceted particles with sharp edges and recessed faces and internal cavity structure (Fig. 2a). Transmission electron microscopy (TEM) image reveals a square/cube-like morphology with homogeneous contrast and clean outlines (Fig. 2b). Elemental mapping analysis determined the uniform distribution of the Cu and S on the CuS (Fig. S3). X-ray diffraction (XRD) patterns exhibit the expected reflections of hexagonal CuS with minimal impurity peaks; the control pattern for CuO is shown for comparison (Fig. 2c). The phase purity of CuS matters because electronic structure governs photothermal and charge-transport behavior. Ultraviolet–visible (UV–vis) spectra demonstrates that CuS absorbs strongly across the UV–visible region and notably at 355 nm, whereas CuO absorbs less strongly (Fig. 2d). CuS is known to host high hole concentrations due to copper vacancies, enabling localized surface plasmon–like absorption in the visible/near-IR and efficient photothermal conversion [19]. Although the plasmon center of CuS typically occurs at longer wavelengths, the measured absorbance at 355 nm is still higher than that of CuO, indicating superior photon capture. Together, XRD and UV–vis indicate that the CuS particles provide an optically and electronically favorable surface for LDI.

Nitrogen adsorption–desorption (BET) analysis shows a specific surface area of 20.489 m^2^/g for the hollow CuS nanocubes, compared with 6.172 m^2^/g for Cu_2_O (Fig. S4). The increased specific surface area of CuS is attributed to its hollow, faceted morphology, providing more exposed active facets and recessed features that enhance analyte-surface interactions and local energy confinement during laser irradiation. Electrochemical support for the proposed interfacial mechanism. To directly probe carrier dynamics at the CuS interface, we performed EIS and transient photocurrent measurements on CuS/FTO films (with CuO/FTO). Nyquist analyses show that CuS exhibits a substantially lower Rct than Cu_2_O (Fig. S5). Photocurrent on/off traces reveal higher steady-state photocurrent and faster rise/decay for CuS, consistent with efficient carrier generation/separation and reduced recombination (Fig. S6). These findings support our interpretation that the p-type, vacancy-rich CuS facilitates rapid interfacial charge transfer during laser excitation, thereby diminishing recombination with nascent analyte ions and lowering the desorption/ionization threshold that underpins the improved small-molecule signals in LDI-MS. In a typical metabolic analysis process, 1 μg of the CuS nanocubes was loaded onto each sample spot formed by 0.1 μL of serum, allowing for the automatic detection of up to 384 microarrayed sample spots with high throughput. The detection can be completed within 30 s per sample.

The performance of the CuS matrix for small molecules in LDI-MS is placed in the context of detection sensitivity, molecular selectivity, analytical reproducibility, and untargeted detection capability. A comparison of the performance with the traditional organic matrix was conducted for evaluation. Four representative metabolites—Suc, Glu, Pro, and Man—were analyzed in ten independent repeats using three matrices: CuS and the two common organic matrices CHCA and DHB (Fig. 2e). The improvement is most meaningful in the low-m/z region, where organic matrices notoriously contribute rich background and abundant clusters, masking small metabolites. For all four metabolites, CuS yields the highest signal intensities (Fig. S7). Limit of detection (LOD) for four metabolites was measured to be 5–50 × 10^−6^ M after successive dilutions (Fig. S8), which is lower than the physiological concentration in serum. Here, CuS provides a cleaner low-mass window and stronger alkali-adduct or protonated signals with low LOD while avoiding low-mass interference, which directly improves sensitivity for metabolite detection.

Repeatability is quantified at two levels. First, Fig. 2f summarizes the individual intensity trajectories over the ten tests and reports the CVs for each metabolite. The CVs remain comfortably within expected metabolomics quality windows, and most analytes show single-digit to low-teens CVs, reflecting stable shot-to-shot behavior of the CuS matrix. These values compare favorably to many reported LDI methods, where day-to-day and spot-to-spot CVs in the 15–30 % range are common for small molecules. The modest dispersion here is consistent with efficient photothermal conversion and a uniform, crystalline particle bed that resists catastrophic hot-spotting (Fig. S9). Before conducting the serum test using the CuS nanocubes, we evaluated their salt tolerance and protein resistance for four metabolites in a high-salt or protein mixture (15 mM Na^+^, 0.5 mM K^+^, and 10 mg/mL bovine serum albumin). These results indicate that the LDI-MS platform can efficiently profile metabolites from biological matrices without complex pretreatment. (Fig. S10). Second, Fig. 2g generalizes reproducibility to complex samples by computing CVs for ∼463 *m*/*z* features detected from a representative serum specimen across 30 independent measurements (drawn from three separately prepared serum replicates). The median CVs across the three serum samples were around 7 %, 14 %, and 13 %, respectively. The shapes reveal that the bulk of features fall within narrow variability bands and that there are few long tails. These medians capture the global precision of the workflow and demonstrate that the method is sufficiently stable for discovery-scale metabolomics. Moreover, the spread of CVs is narrow for the best-performing serum set (median ∼7 %), indicating that, when handling is consistent, the CuS matrix supports exceptionally tight technical precision.

Mechanistically, the superior performance of CuS traces to coupled optical and electronic factors. High absorbance at 355 nm ensures robust photon capture [20]. The faceted geometry and recessed features enhance near-field concentration and thermal confinement at the surface, while the p-type, vacancy-rich electronic structure supports the rapid movement of photogenerated carriers, reducing recombination with nascent analyte ions [15]. In LDI terms, this means a lower fluence threshold for desorption, shorter residence time for excited electrons/holes in the interfacial region, and more stable [M+Na]^+^, [M+K]^+^, or [M+H]^+^ formation [21]. In contrast, the weaker absorption and different band structure of Cu_2_O result in less efficient photothermal conversion at 355 nm and poorer charge transfer, explaining its inferior performance in preliminary tests [22]. Relative to organic matrices, CuS avoids matrix-derived fragments and clusters in the *m*/*z* < 500 range, directly improving detectability for small metabolites such as amino acids, sugars, and organic acids.

### 3.2 SMP acquisition of TBI by CuS–assisted LDI MS

**Fig. 3:**
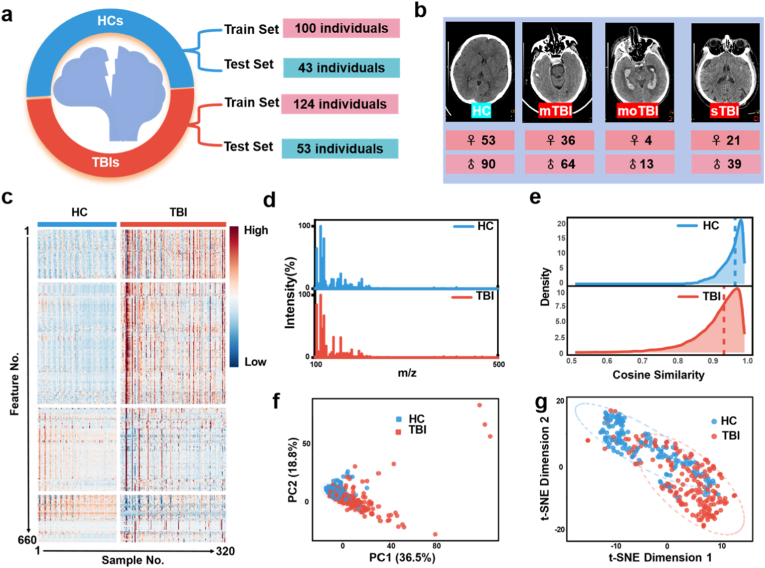
Acquisition of SMPs for TBI based on CuS-enhanced LDI-MS. a) The cohort design for the train set and test set, including 143 HCs and 177 TBIs. **b)** The typical radiological images from HC, mTBI, moTBI, and sTBI with distribution of gender. **c)** The heatmap of the 320 SMPs, each of which contained 660 *m*/*z* features. **d)** Typical MS spectra from the serum samples of HC and TBI at *m*/*z* of 100–500. **e)** The frequency distribution of the similarity scores in the HC group and TBI group, and over 95 % of samples shared similarity scores over 90 % in both groups, displaying the high quality and consistency of the metabolic data. **f-g)** Dimensionality reduction chemometric analysis for discriminating the SMPs. PCA, principal component analysis, t-SNE, T-distributed stochastic neighbor embedding.

We next set out to construct a TBI diagnostic platform based on CuS–assisted mass spectrometry, aiming to elucidate the clinical utility of serum metabolites for TBI diagnosis. Using CuS as the matrix, serum metabolic profiles were acquired by LDI-MS in positive-ion reflection mode from 177 TBI patients and 143 healthy controls (Fig. 3a). Furthermore, the samples were randomly divided into a train set and a test set at a ratio of 7:3 (100 HCs/43 HCs and 124 TBIs/53 TBIs, respectively). This split provides sufficient power for model training yet leaves a meaningful number of held-out cases for generalization checks. Radiological images largely support the diagnosis of TBI patients, and the grading is based on the GCS, with 100 mild cases (mTBI, GCS: 13–15), 17 moderate cases (moTBI, GCS 9–12), and 60 severe cases (sTBI, GCS 3–8) (Fig. 3b). One-way ANOVA and χ^2^ tests indicated no significant differences in age or gender between TBI and HC groups (P > 0.05) (Table S1). Without the need for costly and time-consuming pretreatment, a minute volume of biofluid (≈1 μL) was mixed directly with the matrix, allowing for the collection of low *m*/*z* features (<500 Da). Specifically, the MS data acquisition for the 320 samples was completed within 2 h, and on a 384-spot chip, significantly reducing batch-to-batch variations. A heatmap of the 320 SMPs, comprising 660 *m*/*z* features from TBI and HC samples, is shown in (Fig. 3c). The samples are grouped by class (HC left, TBI right). Coherent blocks of increased (red) or decreased (blue) intensity are visible in the TBI columns relative to HC, indicating systematic, multi-analyte shifts rather than isolated outliers. Equally important is the banding pattern within each class: repeated motifs appear across many samples, a visual cue of technical consistency that aligns with the reproducibility metrics established for the CuS matrix. Relative intensity differences between TBIs and HCs suggest group-dependent metabolic alterations. Fig. 3d contrasts typical single-spectrum profiles from HC and TBI. While both classes show a broad distribution of small-molecule peaks, the TBI trace exhibits altered intensities at multiple *m*/*z* positions, including species that tentatively map to energetic metabolites, amino-acid derivatives. The quantitative backbone for data quality is illustrated in Fig. 3e, which displays the within-class cosine similarity distributions for all pairwise sample comparisons. Both classes center at high similarity (dashed vertical lines near 0.9–1.0), and the HC curve is narrower with a sharper right-tail peak, while the TBI curve is slightly broader. The high within-group similarity means that spectra are reproducible from sample to sample, indicating a strong technical foundation. The greater spread in TBI is expected biologically—TBI is a spectrum disorder with heterogeneous mechanisms (edema, axonal injury, microvascular compromise, neuroinflammation), and this heterogeneity is reflected in metabolite profiles. We employed standard data processing techniques, including baseline correction and normalization, to ensure the comparability of results before further analysis. Unsupervised chemometrics formalize the impression of class structure. In Fig. 3f, principal-component analysis (PCA) separates HC and TBI primarily along PC1 (36.5 %), with additional organization along PC2 (18.8 %). The relatively large variance captured by the first two components suggests that the disease signal is not buried in higher-order noise; instead, a small number of correlated metabolite axes differentiate the groups. Although t-distributed Stochastic Neighbor Embedding (t-SNE) does not preserve global distances, it excels at local neighborhood structure, and here it reveals two partially overlapping but distinct clouds corresponding to HC and TBI (Fig. 3g). The overlap is expected—some mTBI patients present with limited systemic metabolic disturbance, and some HCs inevitably exhibit idiosyncratic metabolic states (diet, circadian variation). Still, the overall separation confirms that class-discriminating information is present independently of any specific modeling assumption, suggesting the need for further machine learning modeling.

### 3.3 Decoding of SMPs for the diagnosis of TBI

**Fig. 4:**
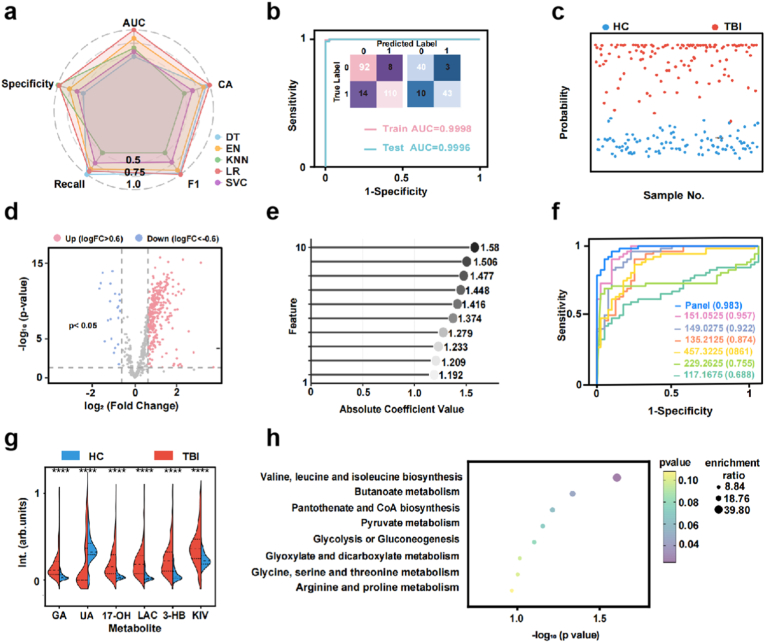
Machine learning based on SMPs achieved precise molecular diagnosis of TBI. a) The comparison of cross-validated area under the curves (AUCs) for the five best-tuned machine learning algorithms in 5-fold cross-validation with 20 rounds, including the SVC (support vector machine), Logistic Regression (LR), K-Nearest Neighbor (KNN), Decision Tree (DT), and Elastic Net (EN). **b)** The receiver operating characteristic (ROC) curves by the LR model for diagnosing TBI in the training set and test set cohorts. **c)** The sample-level plots for distinguishing HC (143 blue dots) from TBI (177 pink dots) in the train set and test set, with a significant difference (p < 0.05) in the predictive probability between TBI and HC. The cut-off value was 0.47. **d)** The volcano plots representing FWER p-values and fold changes (FCs) of the 660 features in the SMPs with criteria (dotted lines) of FWER p-value <0.05 (two-tailed *t*-test, Bonferroni correction) and FC > 1.5 (HC/TBI or TBI/HC), **e)** Absolute coefficient value for the top 10 features from the LR model. **f)** The different ROC curves of panel and feature by the diagnostic LR model for discriminating TBI and HC in the test set. **g)** Violin plot showing the expression levels of identified potential biomarkers for HC and TBI groups. ∗∗∗∗ represented p < 0.0001. **h)** Potential pathways were differentially regulated in HCs and TBI patients. The color and size of each circle were correlated with the p-value and enrichment ratio. Statistical significance was calculated using enrichment analysis by MetaboAnalyst 5.0 (https://www.metaboanalyst.ca/). (For interpretation of the references to color in this figure legend, the reader is referred to the Web version of this article.)

For the SMP-based diagnostic model, we evaluated five representative machine-learning algorithms with different hyperparameter combinations in the discovery cohort. Averaged cross-validated AUCs (5-fold, 20 repetitions) were compared for logistic regression (LR), k-nearest neighbor (KNN), support vector classification (SVC), elastic net (EN), and decision tree (DT). Analysis of the model's performance was carried out in terms of the AUC, classification accuracy (CA), specificity (Sp), recall (Recall), and F1 score (F1). The radar plot shows that the LR algorithm with the best-tuned hyperparameters achieved significantly higher performance than the other four algorithms (p-value <0.001, Fig. 4a, Table S2). This convergence across inductive biases indicates that the diagnostic signal is robust and not an artifact of one algorithm's assumptions. It also suggests a favorable bias–variance trade-off: the data contain low-dimensional structure (as implied by PCA/t-SNE in Fig. 3) that linear margins (LR/SVC) can exploit without overfitting, while non-linear models add little because the separation is already strong. Next, the diagnostic model was trained by SMPs from the train set with the best-tuned LR algorithm. For diagnosing TBI, the LR model achieved high performance with an AUC of 0.9998 (95 % CI: 0.9932–1.000), with a sensitivity of 99.6 % and a specificity of 93.6 %. Notably, in the test set, the high diagnostic performance of the LR model was validated with the AUC of 0.946 (95 % CI of 0.880–1.000), the sensitivity of 90.9 %, and the specificity of 90.5 % (Fig. 4b). The confusion matrix further verified the reliability of the machine learning strategy (Fig. 4b insert). Fig. 4c displays sample-level posterior probabilities from the LR classifier (HC vs TBI) across train and test set. The swarm of individual points shows that most HCs cluster near probability zero and most TBIs near one, with a sharp decision boundary around the reported cut-off (∼0.47). The small overlap band likely reflects borderline biology—mild injuries near the clinical threshold, variable time since injury, or metabolic confounders such as fasting status—rather than model instability. Altogether, the diagnostic model based on SMPs realized a precise, rapid, and non-invasive diagnosis of TBI.

The diagnostic method using the biomarker panel with sufficient information was convenient and cost-effective in clinical practice [23]. To further support the diagnosis of TBI, *m*/*z* features in SMPs were selected to construct a diagnostic feature panel. Firstly, 383 features were identified using volcano plots with a false discovery rate (FWER) p-value <0.05 and a fold change (FC) > 1.5 (Fig. 4d). Many significant features show moderate effect sizes rather than extreme outliers. This breadth enables compact panels to be assembled in multiple ways without compromising performance, thereby improving robustness to unavoidable analytical variation. Then, absolute LR coefficients highlight the ten most influential signals (Fig. 4e and Table S3**)**. Feature selection was performed by fitting logistic regression to the training set, retaining features with P < 0.05 and FC > 1.5, ranking the survivors by absolute standardized LR coefficient, and keeping the top 20 candidates. All combinations of these 20 features were then evaluated by LR, and the 6-feature panel that yielded the highest AUC (with preference for better calibration and lower inter-feature correlation) was chosen and subsequently tested on the held-out set. In Fig. 4f and Fig. S11, the ROC performance for the multi-feature panel versus individual features in the train and test set was compared. As expected, the panel (AUC of 0.983 with a 95 % CI of 0.966–0.996) outperforms single markers—combining several moderate, partially correlated signals yields a more stable boundary than relying on any one feature. Still, a few single features retain respectable AUCs (AUC of 0.957 for *m*/*z* of 151.0525, AUC of 0.922 for *m*/*z* of 149.0275), providing attractive targets for orthogonal confirmation or for rapid rule-in adjuncts in streamlined workflows. This hierarchy—panel > best singletons > remaining features—is typical when a disease perturbs connected pathways. The collective readout captures both the magnitude and covariance structure of the metabolic shift, whereas single features capture magnitude only. Subsequently, we further annotated the six metabolites, including L-Lactic acid (LAC), 3-Hydroxybutyric acid (3-HB), Glyoxylic acid (GA), Uric acid (UA), 17-Hydroxypregnenolone sulfate (17-OH), and alpha-Ketoisovaleric acid (KIV), through accurate FT-ICR MS and the human metabolome database (https://hmdb.ca) (Table S4). To substantiate biomarker identities beyond accurate mass and HMDB matching, we acquired LC-MS/MS spectra for the key diagnostic features. And a summary table (precursor *m*/*z*, RT, chemical formula, main fragments) is provided in Table S5. Violin plots further illustrated the expression levels of six potential biomarkers with significant differences (∗∗∗∗p value < 0.0001) between the TBI and HCs groups (Fig. 4g). Specifically, five features were over-regulated and one feature was down-regulated in TBI patients. Subsequently, we conducted metabolites set enrichment analysis using the Kyoto Encyclopedia of Genes and Genomes (KEGG) dataset to identify the most strongly influenced metabolic pathways based on the identified metabolites (Fig. 4h and Table S6). The significantly altered metabolites were mainly mapped to central carbon and amino-acid metabolism. The most enriched pathway was valine, leucine and isoleucine biosynthesis, indicating that branched-chain amino-acid (BCAA) homeostasis is markedly perturbed in TBI. BCAAs are tightly linked to mitochondrial energy production and neurotransmitter balance, and their dysregulation is consistent with a shift in systemic energy demand after brain injury. In parallel, enrichment of butanoate metabolism and pantothenate and CoA biosynthesis suggests reprogramming of short-chain fatty-acid turnover and coenzyme A–dependent acyl fluxes, further supporting an injury-induced remodeling of mitochondrial substrate utilization.

Several carbohydrate-related pathways were also over-represented, including pyruvate metabolism, glycolysis or gluconeogenesis, and glyoxylate and dicarboxylate metabolism. These changes imply enhanced glycolytic throughput and engagement of anaplerotic shunts to sustain the tricarboxylic-acid (TCA) cycle under conditions of bioenergetic stress, in agreement with the concept of a “metabolic crisis” in TBI. In addition, the enrichment of glycine, serine and threonine metabolism and arginine and proline metabolism points to alterations in one-carbon metabolism, redox buffering, and nitrogen handling. Together, these pathway signatures reveal a coordinated reprogramming of energy production, amino-acid utilization and intermediary metabolism in TBI, providing a mechanistic context for the serum metabolic fingerprints captured by the CuS-assisted LDI-MS platform.

TBI is a significant public health concern that affects millions of people globally [24]. Current estimates are that TBI causes one-third of global injury-related deaths and disability [25]. TBI is characterized by a “metabolic crisis” that occurs in the brain as a response to injury [26]. There is nearly a 40 % increase in the resting energy expenditure of an injured brain compared to a noninjured brain [27]. The hypermetabolic state is accompanied by increases in lipid peroxidation, as well as hypermetabolism of ketones and hypometabolism of glucose, respectively. Many of these changes can be directly or indirectly observed in the plasma or serum metabolome due to diffusion of metabolites across the blood-brain barrier (BBB) [28]. Meanwhile, serum/plasma samples provide more information about both BBB disruption and the degree of brain cell injury [29]. Thus, in our study, CuS-assisted SMPs, uniting an LR model, provide a robust molecular fingerprint for TBI diagnosis [30]. After machine-learning-based data parsing, we identified the distinctive systemic metabolic reprogramming associated with TBI, proposing an analytical platform and providing a credible, mechanistically anchored route toward deployable serum tests for objective TBI assessment.

### 3.4 Dynamic serum metabolic signatures for TBI severity staging

**Fig. 5:**
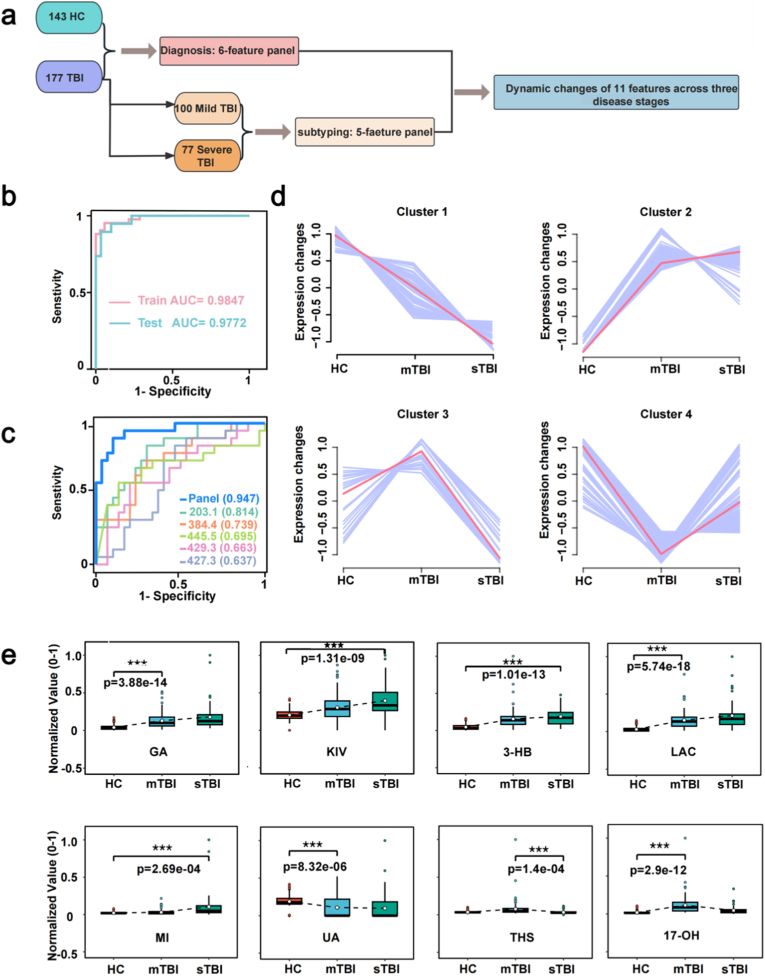
Dynamic Serum Metabolic Signatures for TBI Severity Staging. a) Illustration of discovering TBI potential biomarkers from 143 healthy controls, 100 mild TBI samples and 77 severe TBI patients. **b)** The ROC curves by the LR model for classification of TBI stage in the train set and test set cohort in SMPs, **c)** The ROC curves by the LR model for classification of TBI stage in the test set in 5-features panel, **d)** The clusters each represented a typical type of change trend of the features, **e)** The distribution of 8 features as potential biomarker.

The GCS is the gold standard for diagnosing the severity of TBI [31]. However, the heterogeneity in the progression of TBI among patients often leads to misdiagnosis of severity and inaccurate prediction of outcomes. Metabolomics may enable the quantitative measurement of brain damage, as the concentration and type of metabolites can change in response to the evolving damage [32]. Thus, we investigated dynamic serum metabolic signatures for staging the severity of TBI. Workflow and cohorts for stage-resolved biomarker discovery begin with two groups: 143 HCs and 177 TBI patients (Fig. 5a). For severity, we defined a two-class problem on a severity subset comprising mild TBI (mTBI, n = 100) and severe TBI (sTBI; n = 77). In the present analysis, “sTBI” operationally aggregates individuals with GCS ≤12 after quality control and metadata harmonization; this decision reflects clinical practice where moderate and severe injuries frequently trigger similar management pathways and share molecular signatures of greater systemic disturbance. On this foundation, we derived two compact readouts: a six-feature diagnostic panel (HC vs. TBI) and a five-feature subtyping panel (mTBI vs. sTBI), and we further tracked stage-dependent changes in an extended set of severity-linked features. As LR model demonstrates excellent performance in differentiating between HCs and TBIs, the LR model also offers outstanding performance in distinguishing between mTBI and sTBI both in train set (AUC of 0.9847 with 95 % CI of 0.966–0.996) and test set (AUC of 0.9772 with 95 % CI of 0.943–0.982, Fig. 5b). After the same metabolic feature screening strategy (Figs. S8–9), 5-feature panel accurately separated stages, yielding AUC = 0.947 in the test set and AUC = 0.980 in the train set (Fig. 5c, Fig. S14 and Table S7). Together, these results show that CuS–assisted LDI MS carries sufficient information to separate HC → mTBI → sTBI while allowing parsimony—few features can carry most of the predictive weight.

Unsupervised clustering of the 660 severity-linked features, including 11 candidates, revealed four archetypal trajectories across HC→mild→severe. Cluster 1 decreases monotonically (consistent with consumption or pathway inhibition); Cluster 2 increases monotonically (consistent with accumulation or up-regulated synthesis); Cluster 3 shows an inverted-V (peaking in mTBI then declining—an acute response that blunts with severity); and Cluster 4 shows a V-shape (dipping in mTBI and rebounding in sTBI—suggestive of compensatory overshoot) (Fig. 5d). These patterns indicate heterogeneous metabolic remodeling with progression and pathways move in distinct temporal/severity modes—some cumulative (monotone), others transient (non-monotone). This heterogeneity accords with modern views of TBI as a spectrum disorder with overlapping axes of bioenergetic stress, neuroinflammation, and membrane remodeling. Violin distributions for 11 candidates show stepwise shifts across groups, consistent with the cluster trends, supporting their use as stage-informative biomarkers (Fig. 5e, Fig. S15). Cluster 1 showed a monotonic decrease and included UA and 7-dehydrocholesterol (7-DH), consistent with progressive loss of antioxidant and sterol pools. Cluster 2 displayed a monotonic increase and comprised GA, KIV, 3-HB, LAC, myo-inositol (MI), tetrahydrodeoxycortisol (THS), 17-OH, and desmosterol (DES), reflecting escalating disruption of central carbon, osmolyte, and steroid metabolism with severity. Cluster 3 captured inverted-V patterns (features peaking in mTBI and declining in sTBI; not all shown), whereas Cluster 4 exhibited V-shaped behavior, exemplified by glutathione (GSH), which dipped in mTBI and partially recovered in sTBI. Together, these clusters delineate monotone and non-monotone modes of metabolic remodeling across the HC → mTBI → sTBI spectrum.

CuS-assisted SMPs encode robust information about TBI severity. A simple LR classifier distinguishes mTBI from sTBI with high accuracy (test AUC 0.9772), and a compact five-feature panel preserves strong performance (AUC 0.947). Unsupervised clustering reveals four archetypal response modes across HC→mild→severe, reflecting cumulative and compensatory biology. Together, these results support a practical and interpretable framework for severity staging that complements imaging and clinical scales, laying the groundwork for longitudinal monitoring and multi-center translation.

## 4 Conclusions

In conclusion, we report an inorganic-matrix LDI-MS platform that hollow CuS nanocubes to deliver rapid, reproducible, and background-sparse serum metabolomics for TBI. CuS nanocubes suppresses low-m/z matrix noise and improves small-molecule signals compared to CHCA/DHB and CuO. With ∼100 nL serum per spot and <30 s per sample, the workflow supports actual high throughput. On a cohort of 177 TBI and 143 HC samples, SMPs combined with a tuned logistic-regression model achieved high diagnostic accuracy (AUC = 0.946 in test set). A six-feature biomarker panel outperformed any single marker and remained compact enough for translation and orthogonal confirmation. Beyond diagnosis, severity grading (mild vs severe) was robust (test AUC = 0.977; five-feature panel AUC = 0.947), and unsupervised trajectory clustering revealed monotone and non-monotone metabolic response modes across HC→mild→severe in central carbon and amino-acid metabolism, consistent with altered energy production and nitrogen handling in TBI. These trajectories map onto bioenergetic stress, lipid remodeling, and inflammatory signaling. Taken together, CuS-assisted LDI-MS provides a fast, low-sample, and reproducible route to serum-based molecular fingerprints that can enable objective diagnosis, and support severity staging with small, interpretable panels. Future work will validate panels prospectively across centers, refine timelines relative to injury, and integrate orthogonal quantitation to finalize deployable assays for point-of-care TBI assessment.

## CRediT authorship contribution statement

**Lei Shi:** Writing – original draft, Data curation. **Ping Yuan:** Writing – original draft, Funding acquisition. **Jiaxin Hou:** Writing – original draft, Methodology, Data curation. **Junxi Pan:** Data curation. **Kejia Cao:** Writing – original draft. **Yanhui Wang:** Writing – original draft, Methodology. **Si Cheng:** Data curation. **Xuting Shen:** Data curation. **Yongli Yang:** Data curation. **Nengrui Guo:** Data curation. **Yizhen Pan:** Data curation. **Rongxin Li:** Writing – review & editing, Funding acquisition. **Weian Yuan:** Supervision. **Lijun Bai:** Supervision.

## Declaration of competing interest

The authors declare that they have no known competing financial interests or personal relationships that could have appeared to influence the work reported in this paper.

## Data Availability

Data will be made available on request.
