# Supplementary material for: Hollow CuS nanocubes enhance serum metabolic profiles for rapid diagnosis and severity grading of traumatic brain injury
Source: Mater Today Bio. 2025 Nov 21;35:102586. doi: 10.1016/j.mtbio.2025.102586 (PMC12702368; doi:10.1016/j.mtbio.2025.102586)
Supplement: Multimedia component 1 [file mmc1.docx]

Supporting Information

**Hollow CuS nanocubes enhance serum metabolic profiles for rapid diagnosis and severity grading of traumatic brain injury**

*Lei Shi^#1,2,9^,Ping Yuan^#3^,Jiaxin Hou^#4^, Junxi Pan^5^, Kejia Cao^6^, Yanhui Wang^4^, Si Cheng^2^, Xuting Shen^2^, Yongli Yang^1^, Nengrui Guo^1^, Yizhen Pan^1^, Rongxin Li*^4,6^, Weian Yuan*^7^, Lijun Bai*^1^*

1. **Supplemental figure**


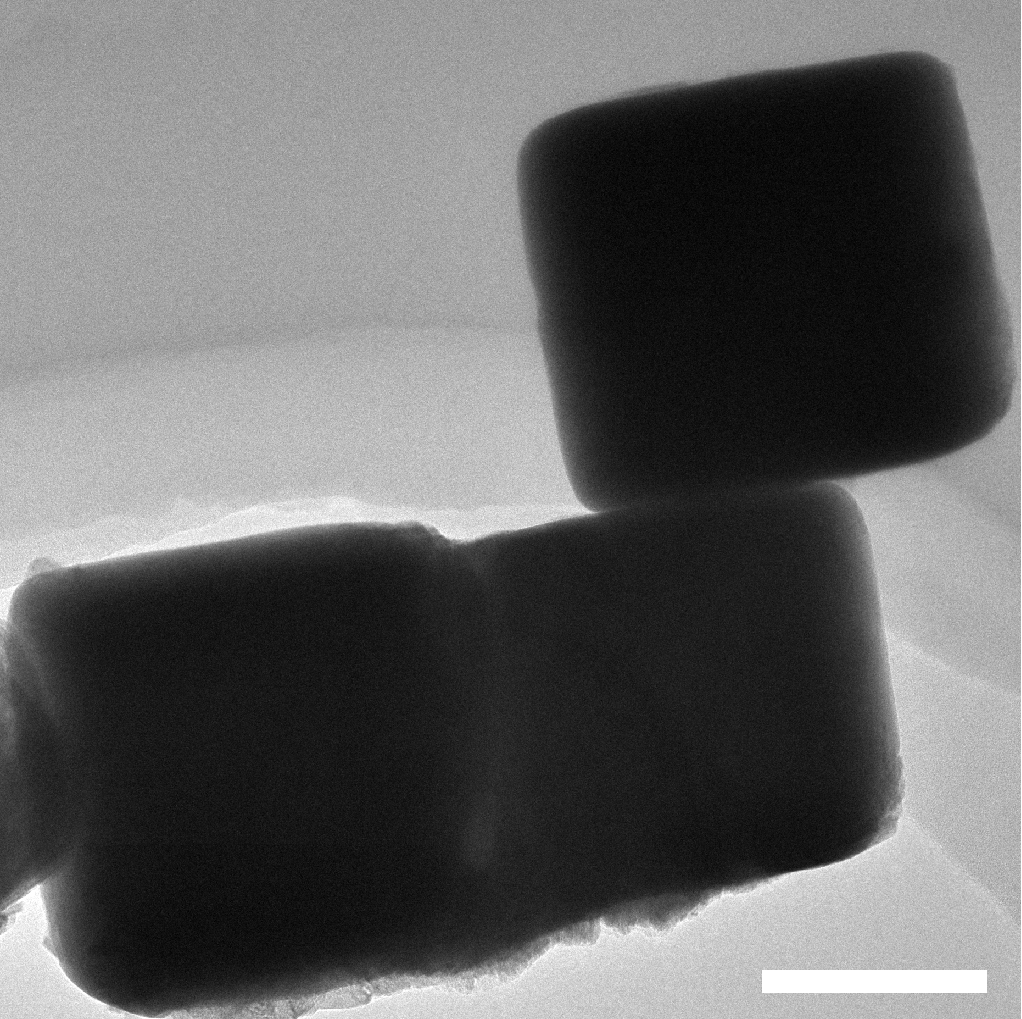


**Figure S1.** The transmission electron microscope image of the Cu_2_O (scale bar of 200 nm).


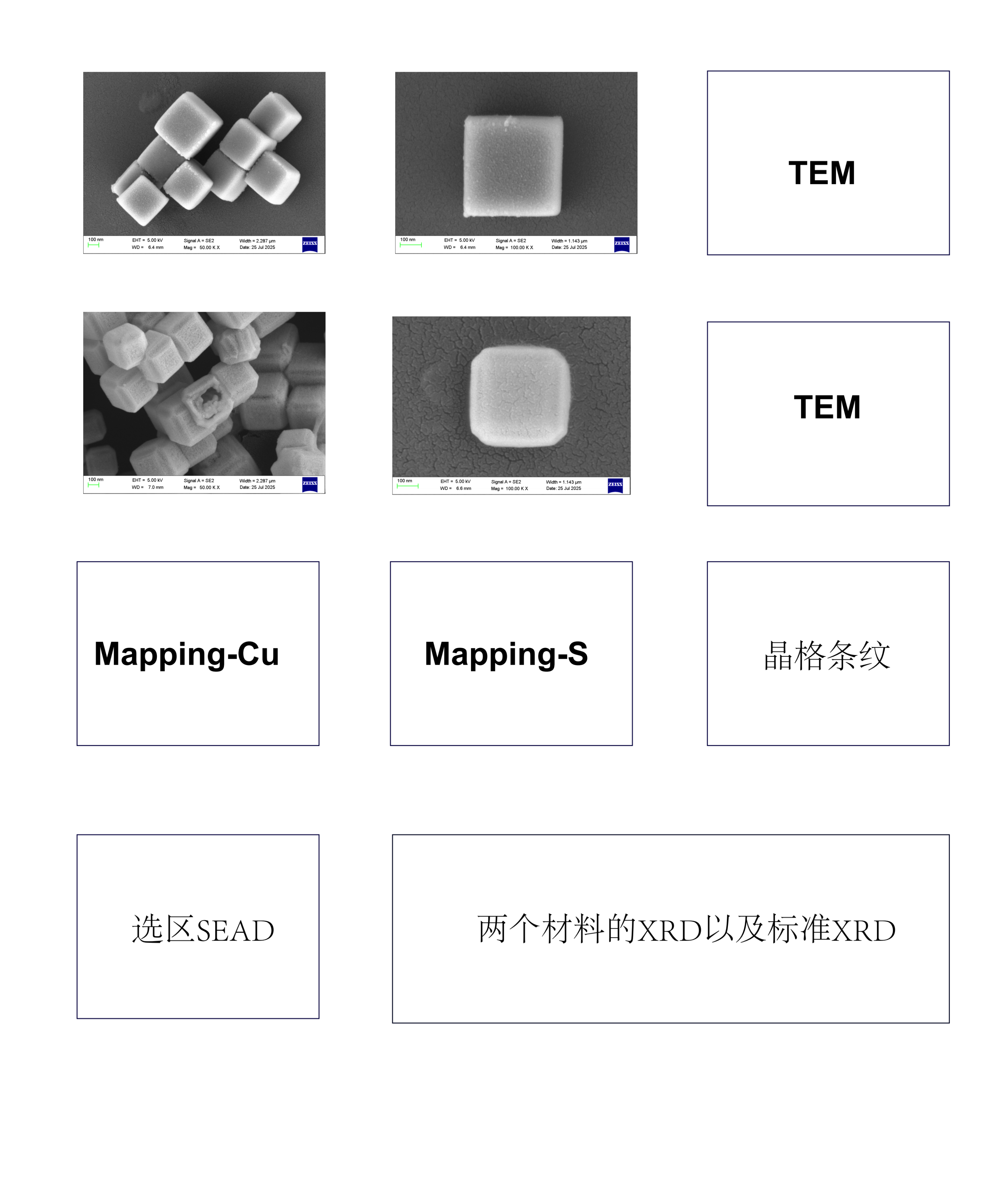


**Figure S2.** The scanning electron microscopy image of the Cu_2_O (scale bar of 200 nm).


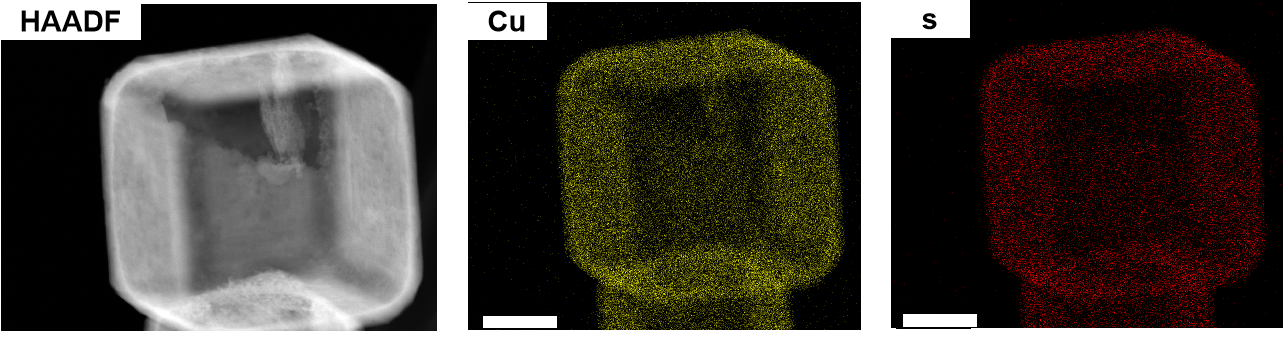


**Figure S3.** The mapping of the CuS (scale bar of 200 nm).


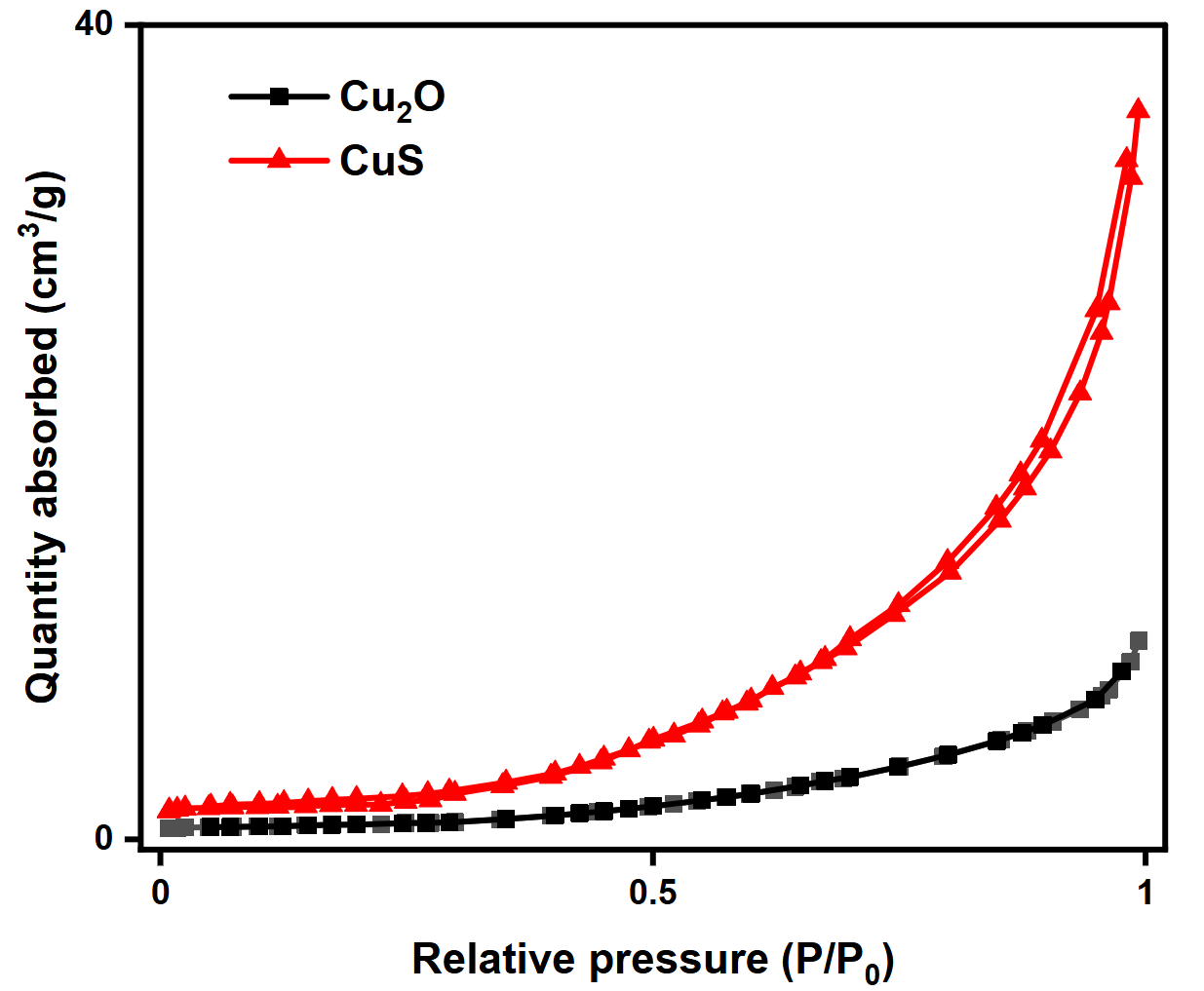


**Figure S4.** Nitrogen adsorption isotherm of CuS and Cu_2_O.


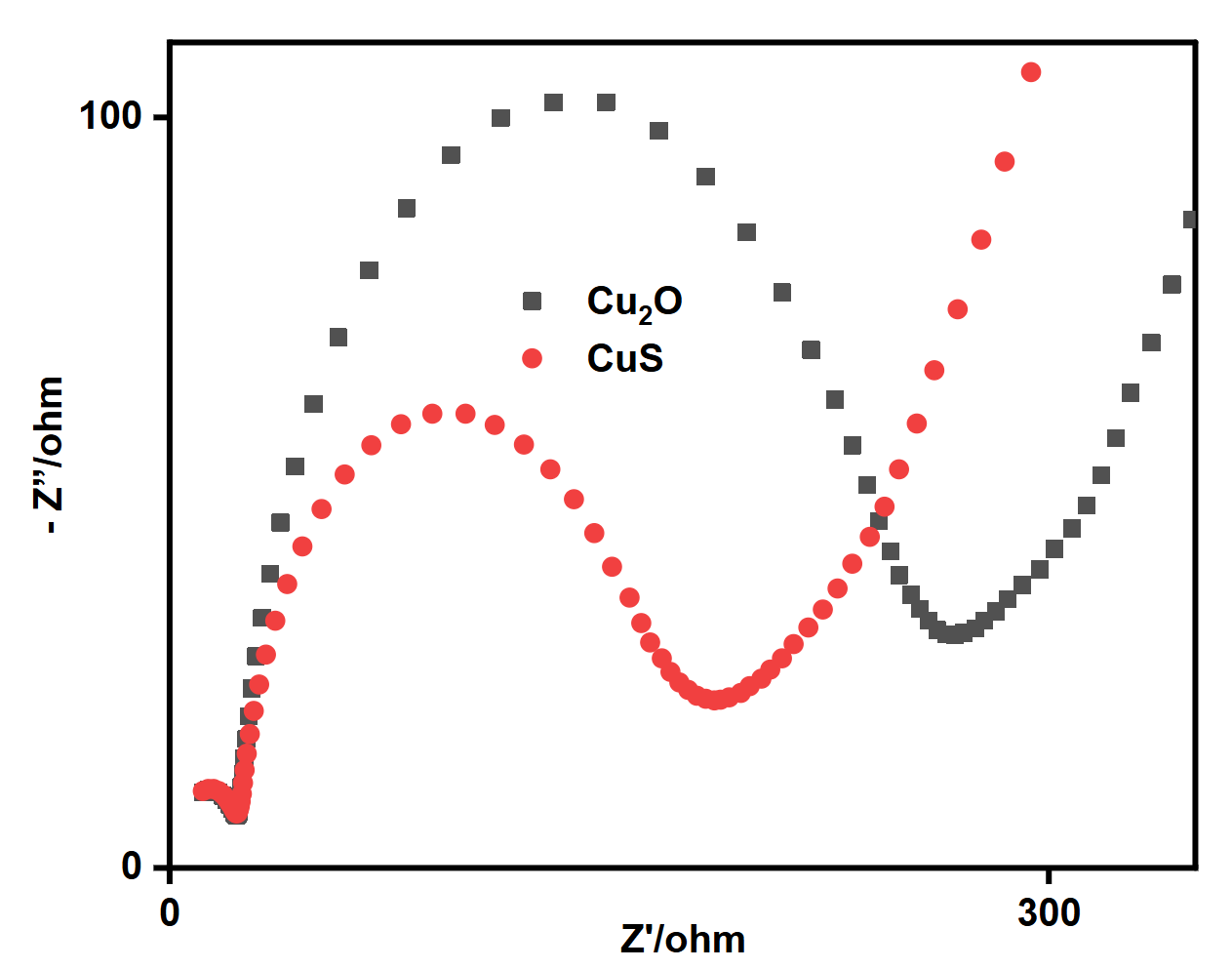


**Figure S5.** Nyquist plots of CuS and Cu_2_O.


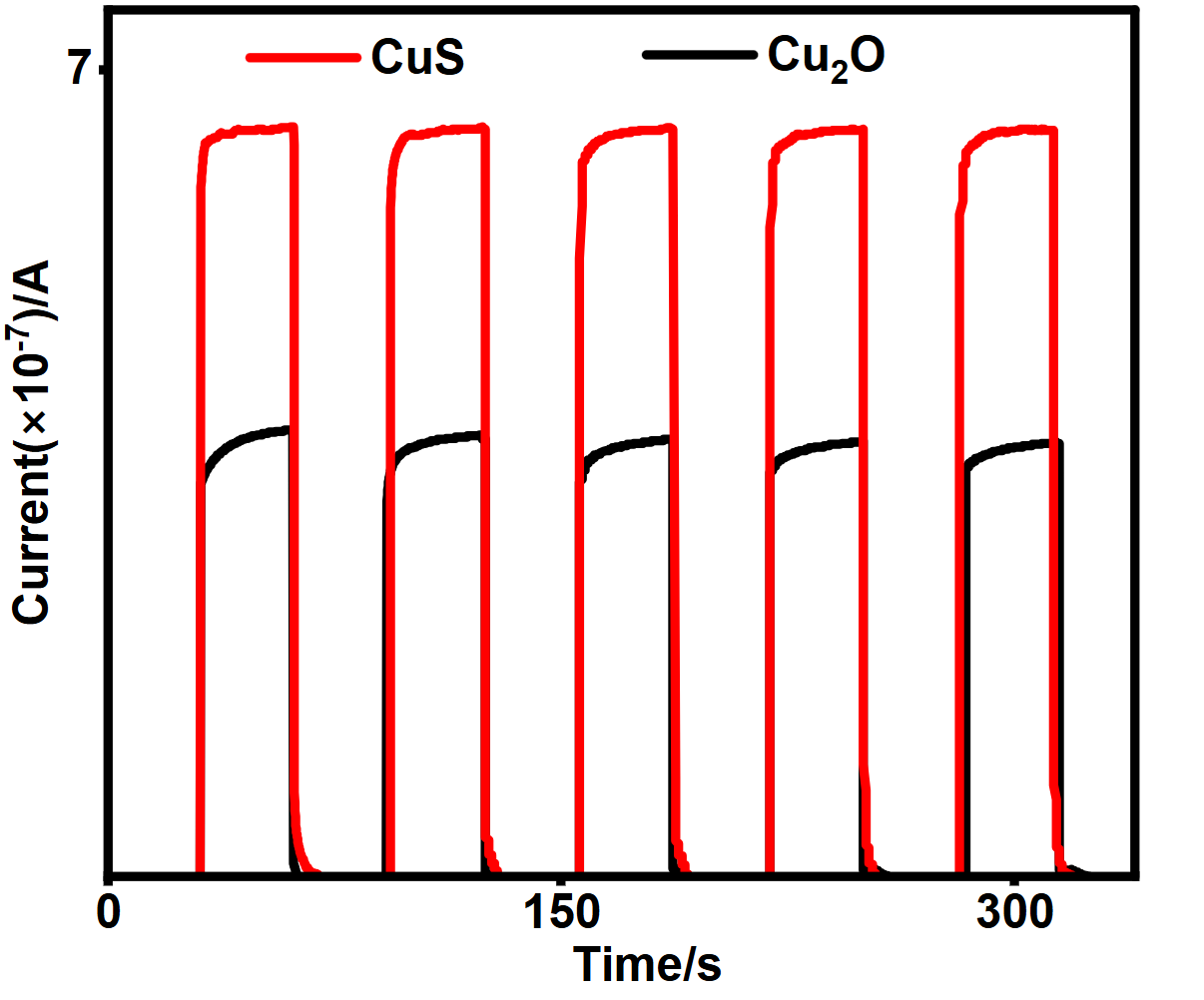


**Figure S6.** Photocurrent-time curves of CuS and Cu_2_O.


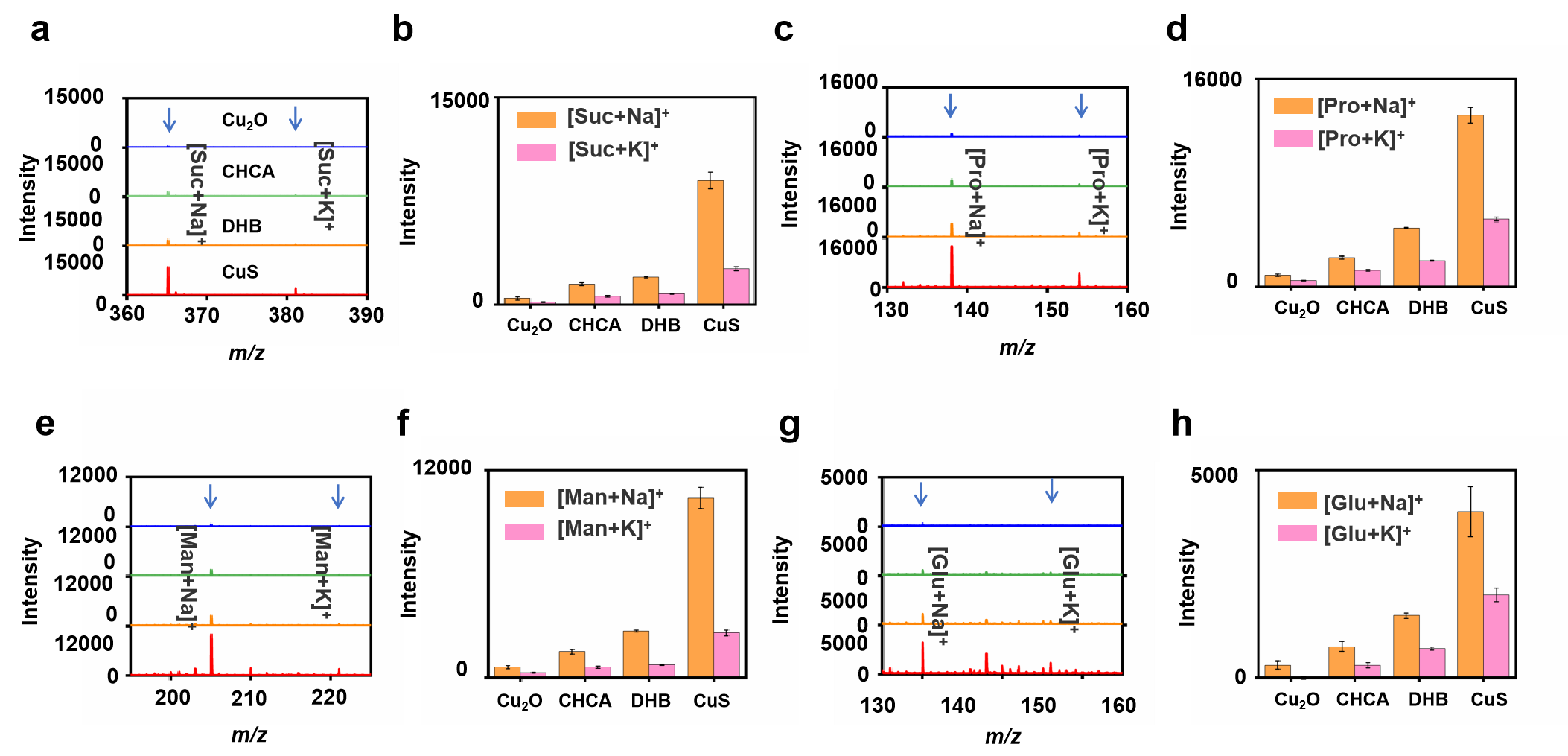


**Figure S7.** MALDI-MS detection for 1mg ml^-1^ sucrose (in a-b), proline (in c-d), mannitol (in e-f), and glutamic acid (in g-h) using Cu_2_O, CHCA, DHB, and CuS as matrix.


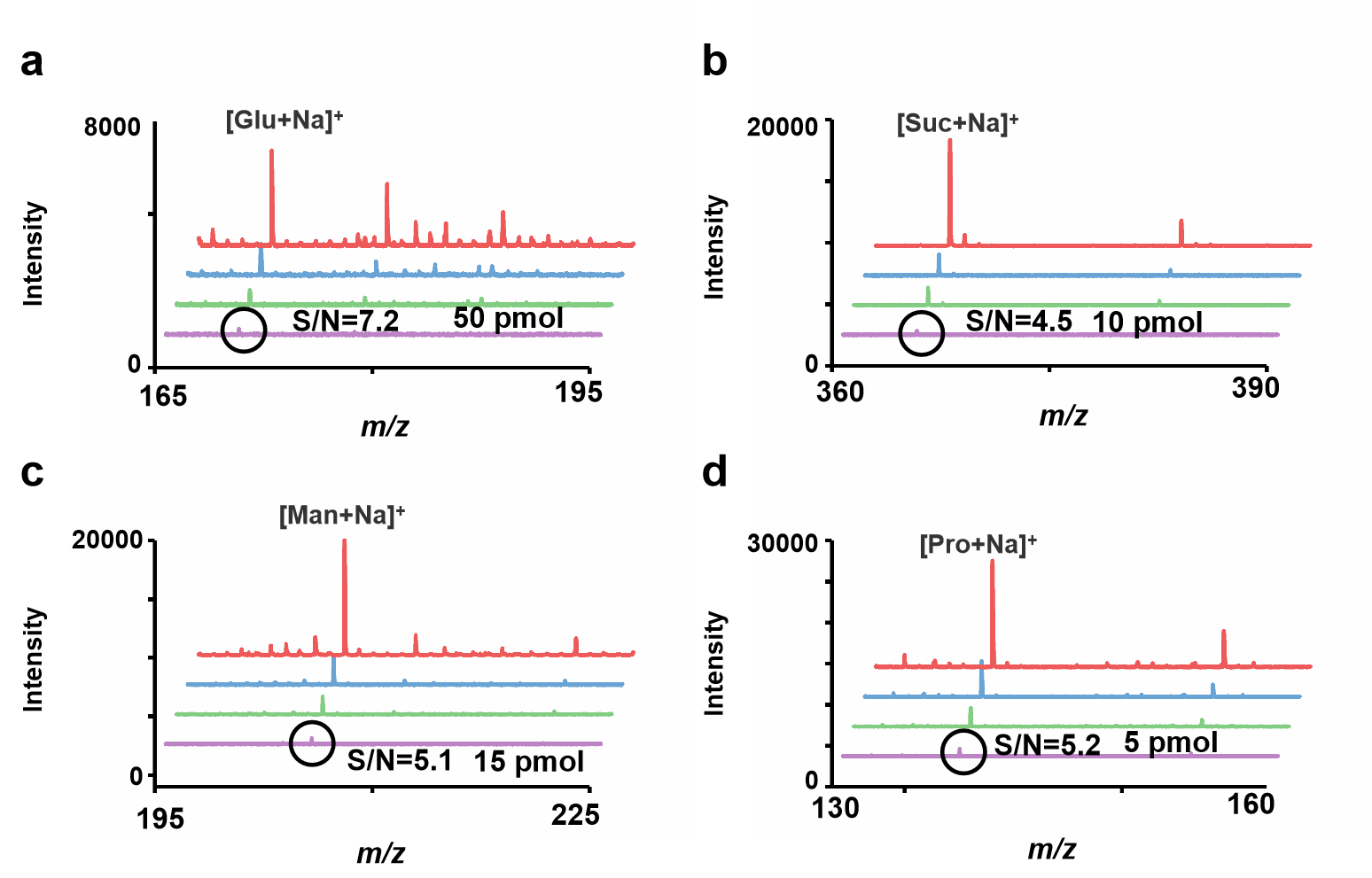


**Figure S8.** The LOD of small molecules.


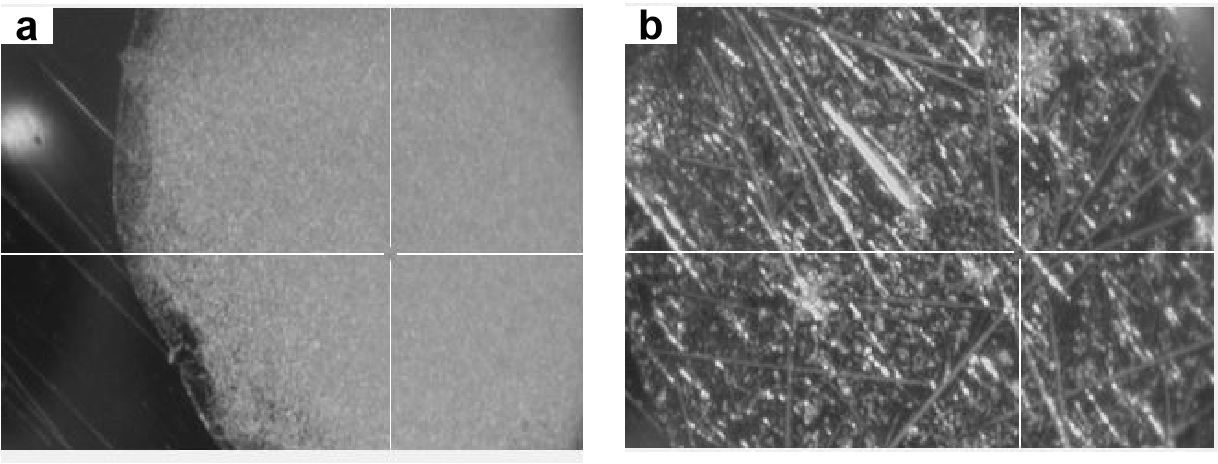


**Figure S9.** Homogeneity of different matrix. a) Hollow CuS nanotubes. b) DHB.


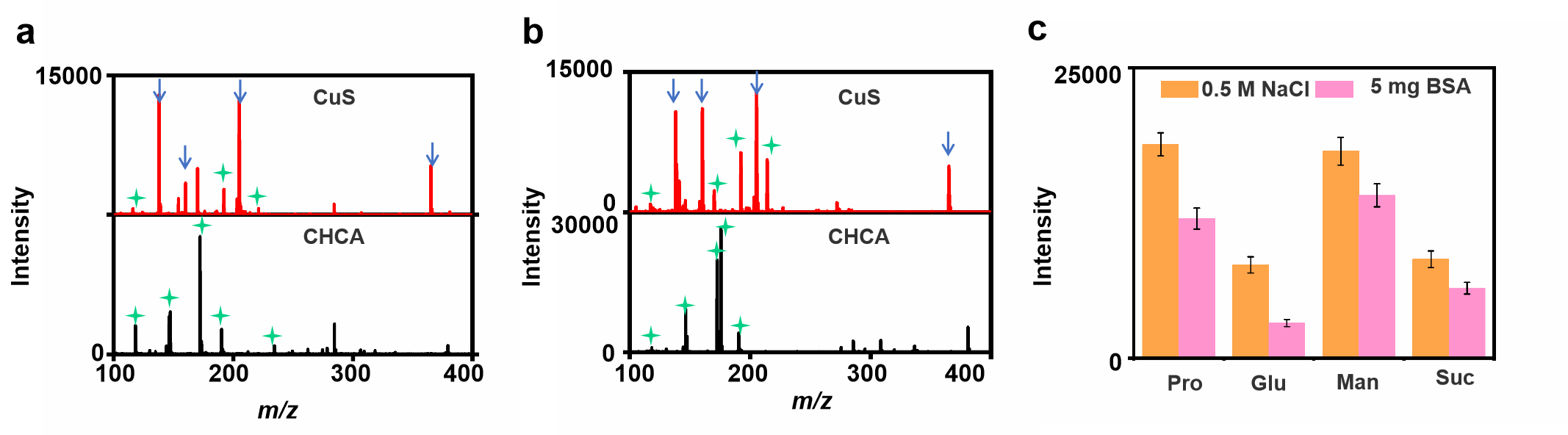


**Figure S10.** Salt and protein tolerance performance. MALDI-MS detection for a mixed solution of four small molecules with high salt (a) and protein (b) (15 mM of Na^+^, 0.5 mM K^+^, aand 10 mg mL-1 of protein of bovine serum albumin).

**
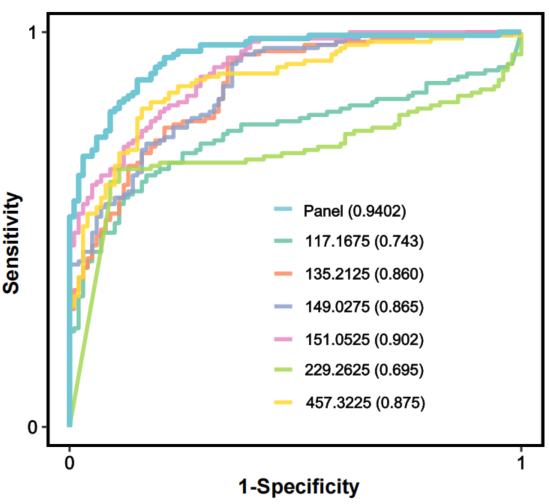
**

**Figure S11.** The different ROC curves of panel and feature by the diagnostic LR model for discriminating TBI and HC in the train set.

**
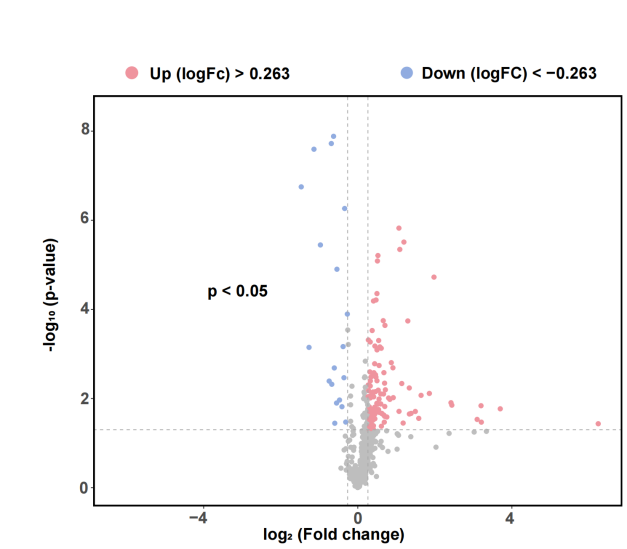
**

**Figure S12.** The volcano plots representing FWER p-values and fold changes (FCs) of the 660 features in the SMPs with criteria (dotted lines) of FWER p-value < 0.05 (two-tailed t-test, Bonferroni correction) and FC > 1.2 (mTBI/sTBI or sTBI/mTBI)

**
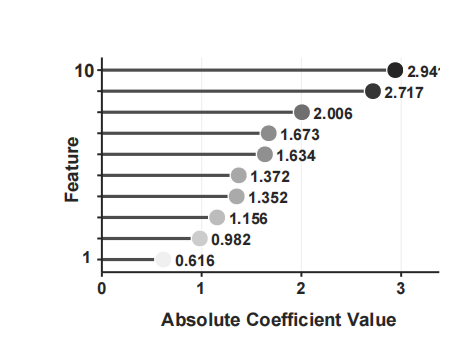
**

**Figure S13.** Absolute coefficient value for the top xx features from the LR model based SMPs of mTBI and sTBI.

**
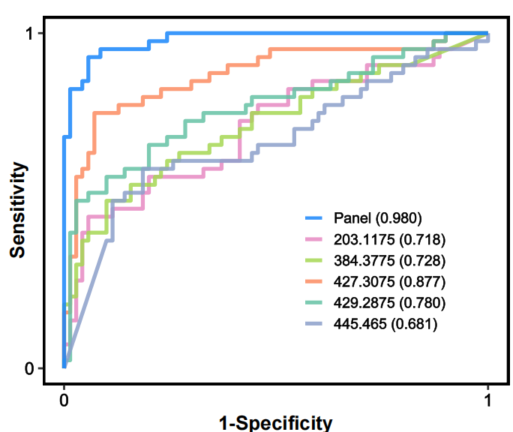
**

**Figure S14.** The different ROC curves of panel and feature by the diagnostic LR model for classification of TBI stage in the train set.


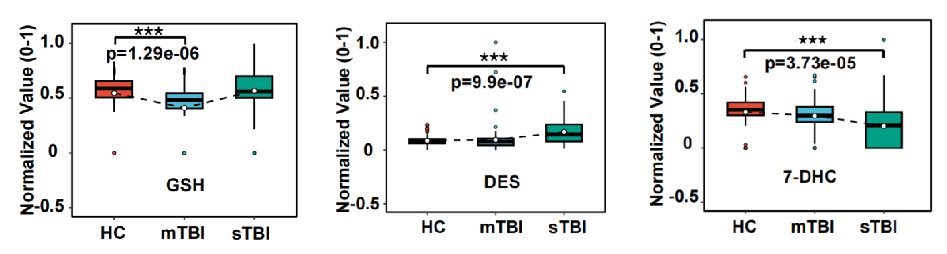


**Figure S15.** The distribution of 3 features as potential biomarker.

**2. Supplemental table**

**Table S1.** **Demographic characteristics of the participants in the main cohort (n = 125).**

|  | Myopic macular degeneration grades | | | |
| --- | --- | --- | --- | --- |
|  | HCs | mTBI | sTBI | P-value^a)^ |
| Num | 143 | 100 | 77 |  |
| Age, years, ± SD | 90/53 | 64/36 | 51/26 | 0.885 |
| Sex, male/female^b)^ | 47.9+15.0 | 50.2+15.1 | 52.4+10.8 | 0.061 |

^a)^ Comparison among groups was analyzed using the one-way analysis of variance (continuous data) or *χ*2 test (categorical data).

^b)^ Sex referred to the sex assigned at birth.

**Table S2. The diagnostic performance of machine learning models based on SMPs in test set.**

| Model | AUC  (95% CI) | Specificity | CA | Recall | F1 |
| --- | --- | --- | --- | --- | --- |
| LR | 0.949 | 0.912 | 0.867 | 0.830 | 0.871 |
| SVC | 0.868 | 0.855 | 0.795 | 0.745 | 0.798 |
| KNN | 0.882 | 0.916 | 0.759 | 0.636 | 0.743 |
| EN | 0.917 | 0.879 | 0.841 | 0.810 | 0.846 |
| DT | 0.850 | 0.836 | 0.852 | 0.865 | 0.864 |

**Table S3. The ten most influential features based on absolute LR coefficients.**

| Rank | 1 | 2 | 3 | 4 | 5 |
| --- | --- | --- | --- | --- | --- |
| Feature | 229.2625 | 474.4 | 329.4325 | 445.465 | 135.2125 |
| Rank | 6 | 7 | 8 | 9 | 10 |
| Feature | 425.44 | 457.3225 | 129.2275 | 467.92 | 103.645 |

**Table S4. The six features in the panel for discriminating the HC group and the TBI group.**

| No. | m/z in  MALDI-MS | Metabolite | Ion adduction | Fold change^a)^ | P-value^b)^ |
| --- | --- | --- | --- | --- | --- |
| 1 | 151.0525 | L-Lactic acid | [M+Na+K-H]^+^ | 4.59 | 1.7e-16 |
| 2 | 149.0275 | 3-Hydroxybutyric acid | [M+2Na-H]^+^ | 2.88 | 3.4e-16 |
| 3 | 135.2125 | Glyoxylic acid | [M+Na+K-H]^+^ | 2.68 | 6.2e-13 |
| 4 | 457.3225 | 17-Hydroxypregnenolone sulfate | [M+2Na-H]^+^ | 2.91 | 6.8e-11 |
| 5 | 229.2625 | Uric acid | [M+Na+K-H]^+^ | 0.59 | 3.8e-5 |
| 6 | 117.1675 | alpha-Ketoisovaleric acid | [M+H]^+^ | 1.65 | 2.0e-10 |

^a)^ Calculated by the ratio of the HC group to the simple TBI group.

^b)^ Calculated by two-tailed t-test.

**Table S5. The LC-MS/MS validation for the biomarker panel for discriminating the HC group and the TBI group**

| No. | Metabolite | Chemical formula | Ion adduction in LC-MS/MS | Retention  time (sec) | m/z in LC-MS/MS | Main MS/MS fragments |
| --- | --- | --- | --- | --- | --- | --- |
| 1 | L-Lactic acid | C_3_H_6_NO_3_ | [M-H]^+^ | 161 | 89.024 | 41.0, 43.0, 45.0, 71.0, 87.0, 89.0 |
| 2 | 3-Hydroxybutyric acid | C_4_H_8_O_3_ | [M-H]^+^ | 224 | 103.040 | 41.0, 59.0, 103.0 |
| 3 | 17-Hydroxypregnenolone sulfate | C_21_H_32_O_6_S | [M-H]^+^ | 356 | 411.185 | 97.0, 113.0, 179.0, ... ,411.187 |
| 4 | Uric acid | C_5_H_4_N_4_O_3_ | [M-H]^+^ | 230 | 167.022 | 26.0, 41.0, 42.0, 68.0, 69.0, 81.0, 96.0, 97.0, 123.0, 123.0, 124.0, 167.0, 167.0 |
| 5 | alpha-Ketoisovaleric acid | C_5_H_8_O_3_ | [M-H]^+^ | 110 | 115.040 | 42.0, 98.0 |
| 6 | Glyoxylic acid | C_2_H_2_O_3_ | [M-H]^+^ | 62 | 73.030 | 45.0, 73.0 |

**Table S6.** Metabolic pathways that associated with TBI.

| Pathways | -log(p) | FDR | Enrichment |
| --- | --- | --- | --- |
| Valine, leucine and isoleucine biosynthesis | 1.60 | 1 | 39.80 |
| Butanoate metabolism | 1.33 | 1 | 21.23 |
| Pantothenate and CoA biosynthesis | 1.21 | 1 | 15.92 |
| Pyruvate metabolism | 1.15 | 1 | 13.84 |

**Table S7. The five features in the panel for discriminating the mTBI and the sTBI group.**

| No. | m/z in  MALDI-MS | Metabolite | Ion adduction | Fold change^a)^ | P-value^b)^ |
| --- | --- | --- | --- | --- | --- |
| 1 | 427.3075 | Tetrahydrodeoxycortisol | [M+2Na-H]^+^ | 0.65 | 1.3e-8 |
| 2 | 429.2875 | Desmosterol | [M+2Na-H]^+^ | 1.44 | 6.18e-6 |
| 3 | 445.465 | 7-Dehydrocholesterol | [M+Na+K-H]^+^ | 0.68 | 1.3e-2 |
| 4 | 203.1175 | myo-Inositol | [M+Na]^+^ | 2.46 | 1.8e-4 |
| 5 | 384.3775 | Glutathione | [M+2K-H]^+^ | 1.36 | 6.6e-4 |

^a)^ Calculated by the ratio of the mTBI group to the sTBI group.

^b)^ Calculated by two-tailed t-test.
